# Investigation of the oral infections and manifestations seen in patients with advanced cancer

**DOI:** 10.12669/pjms.295.3493

**Published:** 2013

**Authors:** Lihua Xu, Hualin Zhang, Jinsong Liu, Xiaowei Chen

**Affiliations:** 1 LihuaXu, General Medicine Department, First Affiliated Hospital, Wenzhou Medical University, Wenzhou, China.; 2 Hualin Zhang, College of Stomatology, Ningxia Medical University, Yinchuan, China.; 3 Jinsong Liu, Department of Prosthodontics, School and Hospital of Stomatology, Wenzhou Medical University, Wenzhou 325027, China.; 4 Xiaowei Chen, Gastroenterology and Hepatology Department, First Affiliated Hospital,Wenzhou Medical University, Wenzhou, China.

**Keywords:** Oral infection, Advanced cancer, Treatment strategy, Oral health

## Abstract

***Objective:*** A prospective, observational study was undertaken to investigate the epidemiology of oral infection among the patients with advanced malignancies, and to investigate the effects of therapy strategies and risk factors on the incidence of oral infection.

***Methods:*** The patients with advanced malignancies were enrolled into the study. The incidence of oral infection with different malignant tumor groups or different treatment methods and the diagnoses of oral infection were confirmed. Demographic data on age, gender, bed rest time, nutritional status, smoking habit and the presence of oral prosthesis were also recorded.

***Results: ***Oral infection was prevalent in 46% (391/850) of all cancer patients, with the highest rate in oral and maxillofacial cancer group (67%), followed by Hematological malignancy group (58.6%) and other groups (ranging from 43.3% to 35.3%). Oral candidiasis, oral herpes simplex, and oral mucositis were the popular infectious diseases in the patients. Chemotherapy and radiotherapy, especially combined radio- and chemotherapy, resulted in more oral infections compared with palliative care and surgery. Poor nutritional status and oral prosthesis were identified as independent risk factors associated with oral infection.

***Conclusion: ***Oral infection is prevalent among advanced cancer patients and associated with therapy methods and risk factors. More oral health care should be carried out for the patients with advanced malignant tumor.

## INTRODUCTION

According to Chinese cancer registry, more than 2 million Chinese will develop cancer in one year. These newly diagnosed malignancies will include 600,000 lung cancers, 300,000 gastrointestinal cancers, 210,000 breast carcinomas, 90,000 lymphomas, and 72,000 cases of leukemia, etc.^1^ It was reported that about 10–50 % of the patients will develop oral infections.^2^^-^^4^ Oral infections are also frequent complications of cancer treatment.^5^^,^^6^

However, Oral infections caused by cancer and/or its treatment are not well documented. Oral status is often insufficiently documented in the patient’s medical records. It has been claimed that physicians and nurses show less attention to the mouth than to other parts of the body. Oral problems may be underreported by the patients and inadequately addressed by physicians, especially for cancer patients in advanced stage. Both cancer and/or its treatment are able to induce inflammation and damage to the oral tissue. Pain and discomfort are common and can lower intake of fluid and nutrients, which in severe cases can lead to dehydration and malnutrition.

Therefore, it is important to identify and target high-risk cancer patients to implement preventative measures. In our study, we have investigated the incidence of oral infection between cancer groups and treatment methods. The epidemiology, independent risk factors were also described and compared within patients.

## METHODS

This prospective, observational study was performed from January 2006 to December 2010 at the First Affiliated Hospital of Wenzhou Medical College. Inclusion criteria were as follows: patients with ≥16 years of age who were diagnosed with advanced malignant tumor; all patients were required to sign an informed consent form prior to entry into the study. Treatment strategies (palliative treatment, surgery, radiotherapy, chemotherapy, combined Chemotherapy and radiotherapy) of all the included patients were counted. The data on age, gender, nutrition condition, bed rest time, smoking habit, and the presence of oral prosthesis were also recorded. All dentists received training in oral inspection and oral mouth swab technique prior to the study. An oral examination and a swab from the oral cavity were performed by dentists on admission and processed for microbiologic isolation. Symptoms and diagnosis of oral infection were documented.

Oral microbial colonization of the patients were analyzed by using microbial culture techniques. ^7  ^Briefly, all samples collected by swabs were incubated in carbon dioxide culture systems. Brucella blood agar medium was incubated at 37°C in an anaerobic jar for 5 days. The tripticase soy bacitracine vancomycin medium was incubated in 10% carbon dioxide in air at 37°C for 4 days. Identification of possible causative microbes was performed according to methods described by Slots and Reynolds, ^8 ^with commercial micromethod systems for various species of bacteria and yeasts. For the detection of virus, the DNA extraction from the samples was done with the specific primers in a PCR assay described by Thankamani,et al. ^9^^,^^10^

## RESULTS

A total of 850 patients were enrolled into the study. There were 486 (57%) male and 364 (43%) female with a mean age of 48 years (range 16–80 years). The number of every group of cancers and oral infection in the study population are summarized in Table-I. Oral infection was prevalent in 46% (391 ⁄ 850) of all cancer patients. Oral infection varied amongst cancer groups. Oral and maxillofacial tumor patients had the highest rate of infection (67%) followed by Hematological malignancies (58.6%), while other groups had the significantly lower rate of oral infection than former two groups. 

The diagnoses of oral infection showed in Table-II. Among the infection cases, 52% (233/448) of them was diagnosed as oral candidiasis, followed by oral mucositis (20.5%), oral herpes simplex (15.4%) and other diagnoses. 57 patients developed more than one kind of oral infectious diseases. Bacteria were detected in all the patients, while 72% of patients were colonized by fungal, most of them were candida, and 18% of them were detected with herpes simplex virus (HSV) or human papilloma virus (HPV). 

The incidence of oral infection with different treatment interventions were also counted (Table-III).The patients received combined chemo- and radiotherapy showed the highest incidence of oral infection followed by chemotherapy group and radiotherapy group, while surgery group had the lowest infection rate. A logistic regression analysis showed that poor nutritional status and the wearing of oral prosthesis were the independent risk factors associated with oral infection (Table-IV). Other factors such as gender, age, smoking habit (>10cigarettes/day), and long-term bed (>7 days) did not demonstrate a statistical risk for the development of oral infection.

**Table-I:** The incidence of oral infection with different malignant tumor groups

*Malignant tumor groups*	*Number of patients*	*Number of Oral infection*	*The incidence of oral infection (%)*
Tumors of Urinary system	123	49	39.8
Tumors of Gastrointestinal system	152	54	35.5
Tumors of Nerve system	82	30	36.6
Hematological malignancies	128	75	58.6
Tumors of respiratory system	127	55	43.3
Oral and maxillofacial tumors	115	77	67
Tumors of reproductive system	123	51	41.5
Total	850	391	46

**Table-II:** The diagnoses of oral infection

*Diagnoses of oral infection*	*N*	*%*
Periodontal abscess	13	2.9
Necrotic gingivo-stomatitis	10	2.2
Oral candidiasis	233	52
Oral herpes simplex	69	15.4
Oral mucositis	92	20.5
Oral papilloma	10	2.2
Other diagnoses	21	4.7

**Table-III:** The incidence of oral infection with different treatment strategies

*Treatment strategies*	*N*	*Oral infection*	*%*
Palliative care	144	37	25.7
Chemo- and radiotherapy	95	65	68.4
Radiotherapy	225	117	52
Chemotherapy	230	124	53.9
Surgery	156	48	30.7

**Table-IV:** Logistic regression analysis of risk factors for oral infection

*Risk factors*	*P –value*	*Odds ratio*	*95% CI*
Age	0.873	0.93	0.51–1.89
Gender	0.791	0.98	0.71–1.57
Long-term bed(>7 days)	0.815	0.91	0.39–1.87
Oral prosthesis	0.010	1.32	1.34–2.78
Poor nutritional status	0.015	1.02	1.23–1.83
Smoking(>10 cigarettes/day)	0.468	0.83	0.67–1.34

## DISCUSSION

The results of this study demonstrated that the incidence of oral infection in the patients with advanced malignancies was 46%, while other studies ranging from 13% to 52%.^2^^-^^6^There are a number of reasons to explain this disparity, including the diseases spectrum included, the diagnostic criteria used, the diagnostic methods used, and the populations examined in studies. On sub-analysis of our data, it becomes apparent that there is different infection rate among cancer groups. The patients with oral and maxillofacial tumors had the highest incidence of oral infection. Several predictive factors associated with oral infection in oral and maxillofacial cancer patients have been proposed, such as pre-operative chemotherapy or radiotherapy, higher tumor stage, concurrent neck dissection, suture material, prior tracheostomy, etc.^11^^,^^12^

The hematological malignancy group occupies the second highest incidence of oral infection. This is much higher compared with some previously reported studies. ^13^ One reason for the difference could be that our studies selected higher risk patients undergoing mainly conditioning or induction chemotherapy and total body irradiation for hematopoietic cell transplantation. Such treatments are known to lead to damage of the cell-mediated immunity that plays an important role in the pathogenesis of oral infections.^14^ As for other cancer groups, there is much lower infection rate than that of former two groups. The low infection rate may partially be explained by the fact that the patients have a lower degree of generalized immunosuppression.

As for diagnoses of oral infection in our study, oral candidiasis accounted for more than 52%, which was the most popular infectious disease among the patients. The increase in oral candida infection in cancer patients is a well reported finding.^15^Our study indicated that both candida carriage and clinical presentation increased, while the candida carriers need not necessarily manifest clinical disease. In our study, Oral candidiasis is the most common infectious disease for advanced cancer patients, and both *C.albicans* and nonalbicans species are involved in the infection of patients, which has been proved by our microbiologic isolation. The higher rates found in our study could be due to the fact that our patients are mainly in advanced stage of malignant tumor, which resulted in impaired body's general defense system. In addition, our patients are often unable to maintain adequate nutritional status and oral hygiene, in spite of receiving instructions and care.

Bacterial diseases, such as periodontal abscess, oral mucostitis and necrotic gingivo-stomatitis, were also common complications for the patients. There are numerous bacteria which constitute normal oral flora, but which may become pathogenic with immune suppression.^16^Viral infections, such as HSV and HPV, counted for about 18% of oral infection. The hematological malignancy patients with oral ulcers during treatment for hematologic malignancies were easy to get viral diseases. It was reported that in patients receiving radiation therapy for oral and maxillofacial cancer, the prevalence of HSV was near 0%. In patients receiving combined radiation and chemotherapy, however, the prevalence increased to nearly 40%.^17^ These data suggest that immunosuppression due to chemotherapy is the main contributive factor for viral infection on oral cavity for cancer patients. 

As for the treatment methods for advanced cancer patients, the patients received Chemotherapy and radiotherapy, especially for combined Chemo- and radiotherapy, had the higher incidence of oral infection. Chemotherapy is known to lead to damage of the cell mediated immunity which plays an important role in the pathogenesis of oral infection,^14^ while Radiotherapy can lead to mucositis, xerostomia and mucosal damage which promotes manifestation of oral infection.^18^ The incidence of oral infection in Palliative care group and surgery group is the lower than other three therapy methods, but much higher than healthy people. This is partly explained by poor nutritional status and comprised oral health care for advanced cancer patients.

We have also investigated whether age, gender, long-term bed (>7 days), smoking (>10cigarettes/day), nutrition condition and the wearing of oral prosthesis influence the risk of oral infection. Poor nutrition condition was one of risk factor for oral infection, which partly explained by impaired specific and non-specific defences.^19^ The presence of oral prosthesis is the other risk factor for oral infection. Many patients are also oral prosthesis users and the prostheses themselves enhance microbe adhesion and biofilm formation.^20^ Other investigators have also observed an increased risk of oral infections in cancer patients wearing dentures compared with those without. ^21^

In conclusion, the present study shows that patients with oral and maxillofacial cancer and hematological malignancies had the higher oral infection rate than other cancer groups. Oral candidiasis was the most prevalent oral infection followed by oral mucositis and other diagnoses. Chemotherapy and radiotherapy induce the development of oral infection. Poor nutritional status and the wearing of oral prosthesis were the risk factors associated with oral infection.
